# Retinal toxicity caused by hydroxychloroquine in patients with systemic lupus erythematosus: A case report

**DOI:** 10.1097/MD.0000000000025688

**Published:** 2021-06-04

**Authors:** Gang Wang, Ning Zhuo, Zheng Liao, Wei Qi, Feng Tian, Zhenhua Wen, Jingyang Li

**Affiliations:** a Department of Rheumatology and Immunology, Zhuzhou Hospital Affiliated to Xiangya Medical College, Central South University, Zhuzhou; b Department of Nephrology, Xiangya Second Hospital, Central South University, Changsha; c Department of Ophthalmology, Zhuzhou Hospital Affiliated to Xiangya Medical College, Central South University, Zhuzhou, Hunan, China.

**Keywords:** hydroxychloroquine, optical coherence tomography, retinal toxicity, systemic lupus erythematosus

## Abstract

**Rationale:**

Hydroxychloroquine has excellent anti-inflammatory and immunomodulatory effects as one of the antimalarial drugs. In particular, hydroxychloroquine was once widely used as a treatment for the new coronavirus pneumonia epidemic in 2020. Retinopathy caused by hydroxychloroquine is normally irreversible, but little attention has been paid to it.

**Patient concerns:**

A 38-year-old young Chinese woman was taking oral hydroxychloroquine 400 mg daily to control lupus disease activity for six years after the diagnosis of systemic lupus erythematosus (SLE). She did not have any history of eye disease and was admitted to the hospital with a sudden blurring of both eyes.

**Diagnoses:**

The diagnosis of retinal macular degeneration caused by hydroxychloroquine was made after excluding other interfering diseases based on the patient's long-term use of hydroxychloroquine and the results of the eye examination.

**Interventions:**

The patient was discontinued from hydroxychloroquine. To control the recurrence of SLE, she was given intravenous methylprednisolone, oral tacrolimus and mycophenolate. Meanwhile, she was asked to take extra care of her eyes and to come to the hospital every three months to have her vision checked.

**Outcomes:**

The patient's blurred vision improved one week later. Three months later, her vision examination showed no further decline (0.4 in the right eye and 0.6 in the left eye). Meanwhile, the SLE disease activity index (SLEDAI) decreased from six points to five points currently.

**Lessons:**

Retinopathy caused by hydroxychloroquine is irreversible and there is no particularly effective treatment. Discontinuation of hydroxychloroquine, better daily eye protection, and regular vision checks are the keys to preventing retinopathy. Although hydroxychloroquine causing retinal toxicity was mentioned several years ago, the rate and severity of retinal toxicity require further research. How to get more patients to take care of their eyes requires continuous and increased education by doctors.

## 1 Introduction

Systemic lupus erythematosus (SLE) is a chronic autoimmune disease that occurs mostly in young women and causes impaired function of several organs.^[1]^ Hydroxychloroquine is widely used as an antimalarial agent for the treatment of autoimmune diseases because of its good anti-inflammatory and immunomodulatory effects. The incidence of retinal damage has been reported to be about 7.5% with hydroxychloroquine use for more than five years.^[2]^ Patients usually present with normal vision or even no visual symptoms in the early stages. Only a few patients may develop visual field defects when reading. Complete loss of vision may even occur in the advanced stages of the disease.^[3]^ Risk factors for the disease include hydroxychloroquine dose, duration, comorbid renal disease, underlying eye disease, drug-drug interactions, age, and genetic factors. We report here a case of a young female patient with SLE controlled by long-term oral hydroxychloroquine. She had no history of any ocular disease and was admitted to the hospital because of blurred vision in both eyes. She was eventually diagnosed with retinal macular degeneration due to hydroxychloroquine and immediately discontinued hydroxychloroquine and received a hormonal combination with immunosuppressive agents to control lupus disease activity. The patient's subsequent vision examination showed no further loss of vision.

## 2 Case presentation

**Figure 1:**
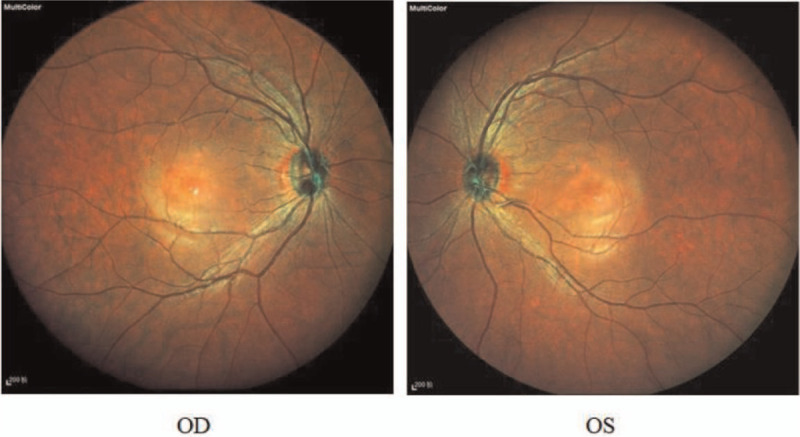
The patient's fundus autofluorescence examination showed reflections in the center of the macula in both eyes, and dotted pigmentation was visible. OD = right eye, OS = left eye.

**Figure 2:**
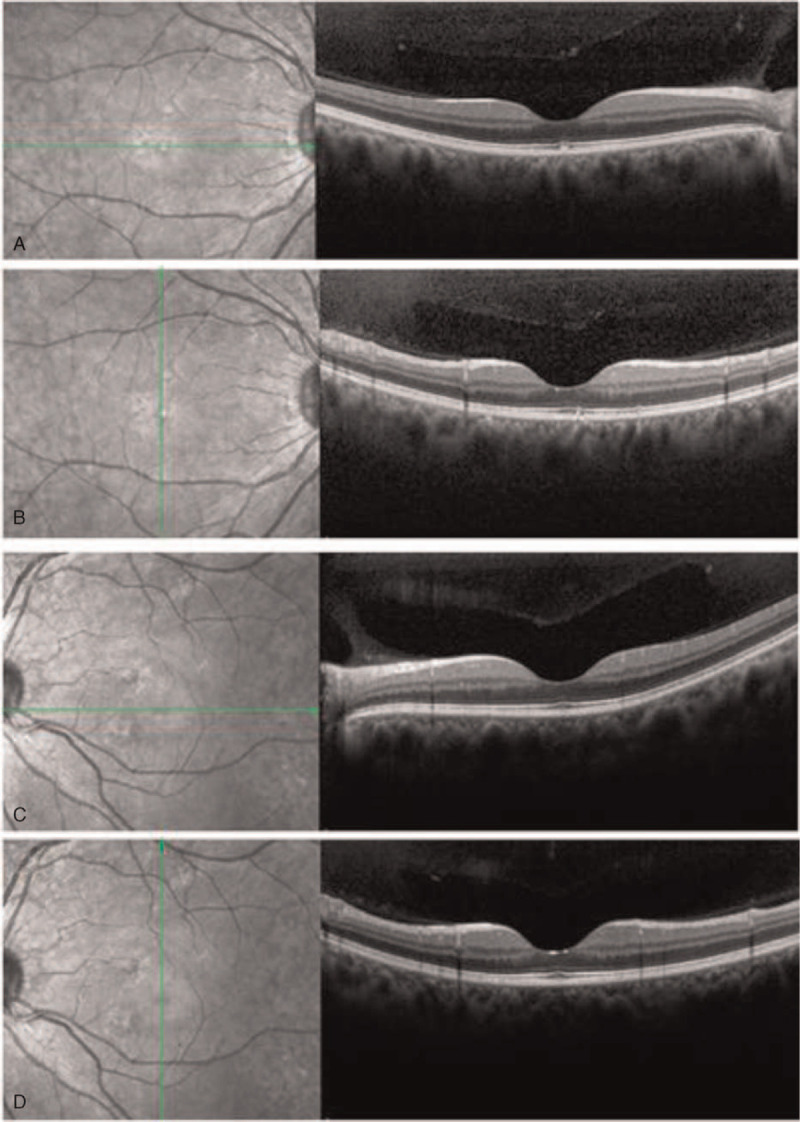
Optical coherence tomography results showed a normal macular physiological curve in the right eye with thickened and elevated submacular retinal pigment epithelium and normal lateral structures (**A, B**). The left eye had normal retinal structure in the central macula as well as thinning of the retinal pigment epithelium on the nasal side of the macula (**C, D**).

**Figure 3:**
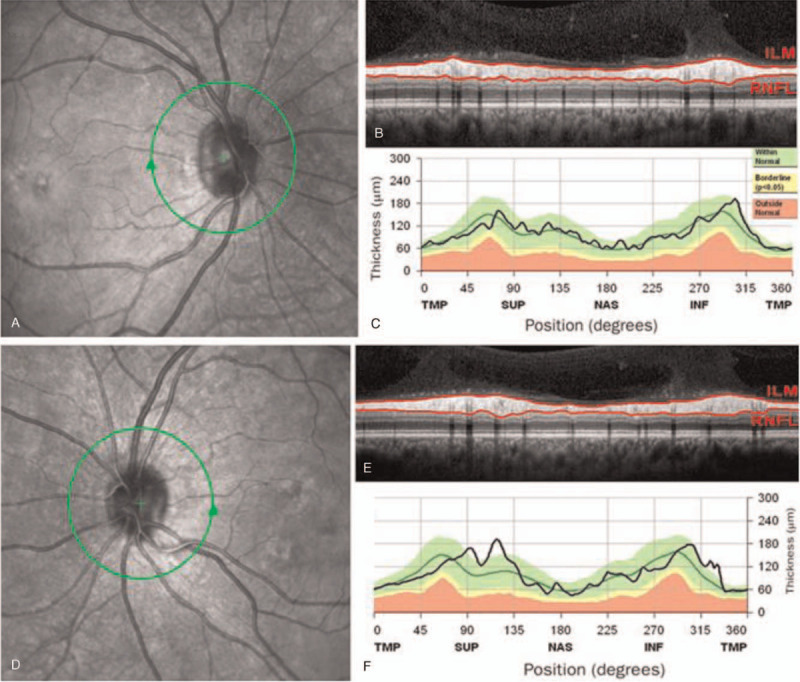
Optical coherence tomography showed normal thickness of the peripapillary nerve fiber layer in the right eye (**A, B, C**) and left eye (**D, E, F**). ILM = internal limiting membrane, INF = inferior, NAS = nasal, RNFL = retinal nerve fibre layer, SUP = superior, TMP = temporal.

A 38-year-old young Chinese woman complained of blurred vision in both eyes. She was diagnosed with SLE six years ago and was taking 400 mg of oral hydroxychloroquine daily to control lupus disease activity. She had no history of any ophthalmic disease and her vision was completely normal six years earlier. Visual acuity examination showed 0.4 in the right eye and 0.6 in the left eye, with an intraocular pressure of 110 mmHg (1 mmHg = 0.133 kPa) in the right eye and 120 mmHg in the left eye. Pupil diameter was normal and direct and indirect pupillary light reflexes were sensitive in both eyes. Laboratory examination showed that white blood cell of 5.28 × 10^9^/L (normal range: 4–10 × 10^9^/L); hemoglobin of 124 g/L (normal range: 110–150 g/L); platelet of 225 × 10^9^/L (normal range: 100–300 g/L); neutrophils of 3.59 × 10^9^/L (normal range: 1.80–6.30 × 10^9^/L); lymphocytes of 1.33 × 10^9^/L (normal range:0.8–4.0 × 10^9^/L), antinuclear antibody 1:160; anti-C1q antibody 11.3 U/ml; 24-hour urine protein 0.04 g; complement C3 0.57 g/L; complement C4 0.08 g/L; erythrocyte sedimentation rate 2 mm/h. Fundus autofluorescence examination showed reflections and dotted pigmentation in the macular centers of both eyes (Fig. 1). Optical coherence tomography showed a normal physiological curve in the macula of the right eye, while the submacular retinal pigment epithelium (RPE) was thickened and elevated but with normal external structure. The retinal structure of the macular center in the left eye was normal, and the pigment epithelium on the nasal side of the macular center was thinned (Fig. 2). The thickness of the nerve fiber layer around the optic disc was normal in both eyes (Fig. 3). Eventually, she was diagnosed with retinal macular degeneration caused by hydroxychloroquine. Hydroxychloroquine was immediately discontinued. To control the recurrence of SLE, she received intravenous methylprednisolone, oral tacrolimus, and mycophenolate. The patient's blurred vision improved one week later. Three months later, her vision examination showed no further decline (0.4 in the right eye and 0.6 in the left eye). Meanwhile, the SLEDAI decreased from six points to five points currently.

## 3 Discussion

Hydroxychloroquine is widely used as an antimalarial drug for its excellent anti-inflammatory and immunomodulatory effects in the treatment of autoimmune diseases. The retinal toxicity caused by hydroxychloroquine is usually irreversible and even progresses after discontinuation of the drug.^[4]^ The mechanisms of retinal toxicity caused by hydroxychloroquine are not well defined and studies on retinal cell metabolism and function are the main areas of focus. Early animal studies found that chloroquine was extensively bound in pigmented ocular tissues (including the RPE, iris, choroid, and ciliary body) of rhesus monkeys on chronic chloroquine administration and eventually accumulated in the retina, which may contribute to retinal toxicity.^[5]^ By the same principle, hydroxychloroquine has a high affinity for melanin-containing skin and ocular tissues and can form irreversible conjugates with melanin in the retinal epithelium, affecting retinal cell metabolism and eventually leading to slowly progressive toxic effects.^[5]^ Hydroxychloroquine and chloroquine can increase the permeability of RPE cells and may also affect the function of RPE cells.^[6]^ Melanin in RPE is able to concentrate hydroxychloroquine and enhance its toxicity. On the contrary, melanin also has a role in helping RPE cells to remove toxic substances.^[7,8]^ Both hydroxychloroquine and chloroquine were found to have a strong inhibitory effect on organic anion-transporting polypeptide 1A2 (OATP1A2) uptake in RPE cells and inhibit the circulating uptake of all-trans-retinal, which suggested that hydroxychloroquine and chloroquine may affect the visual cycle.^[9]^ A recent study found that the toxicity of chloroquine on RPE cells may be related to the downregulation of p150 glued protein expression, which in turn inhibits proliferation and microtubule nucleation in RPE cells.^[10]^ Marmor and Yusuf suggested that hydroxychloroquine affects lysosomal function based on the fact that retinal degeneration caused by hydroxychloroquine occurs in the retinal photoreceptor cell layer, which in turn may affect the autophagic function of the RPE and the stability of the photoreceptor cell membrane leading to retinal toxicity.^[11,12]^ Studies on chloroquine and optic ganglion cells have revealed that the retinal toxicity of chloroquine may affect visual transmission by altering the kinetics of the acid-sensing ion channel 1a (ASIC1a) in optic ganglion cells.^[13]^ However, these studies mainly focused on the acute effects of antimalarials on retinal cells and did not fully reveal the exact mechanism of chronic retinal toxicity.

The earliest changes in hydroxychloroquine retinopathy are pigmented spots at the macula and loss of central recess reflexes. In the later stages of the disease, the area of retinopathy develops into an asymptomatic paracentral dark spot with a distinct area of pigment loss around the central recess, culminating in classic Bull's-Eye Maculopathy.^[3,4]^ Most patients have normal vision or even no visual symptoms in the early stages of the disease. A minority of patients may notice a dark paracentral spot when reading. As the disease progresses, the area of dysfunction expands, the retinal pigment epithelium becomes involved, and the macular lesion invades the central retinal recess, which can eventually lead to loss of vision.^[8]^

Retinal toxicity due to hydroxychloroquine is associated with many risk factors, of which the daily dose of hydroxychloroquine is the most central one.^[2]^ When calculating the dose of hydroxychloroquine, the recommended dose of ≤ 5 mg/kg actual body weight significantly reduces the overall risk of disease.^[14]^ Actual body weight is more vital than ideal body weight. Patients with genetic abnormalities in ABCA4 have been found to be associated with retinal toxicity, while genetic polymorphisms in cytochrome P450 may also be associated with retinal toxicity.^[15,16]^ These genetic factors may explain the differences in the clinical presentation of the disease between European and Asian patients. Besides, the combination of renal insufficiency resulting in decreased creatinine clearance, underlying ocular disease, drug-drug interactions, and age over sixty years are also significant risk factors for retinal toxicity with hydroxychloroquine.

We report here a case of a 38-year-old female patient on long-term oral hydroxychloroquine for control of SLE disease activity. She had no history of any eye disease, yet she was recently admitted to the hospital with blurred vision in both eyes. There are some lessons and insights to be learned from this case. Retinal toxicity caused by hydroxychloroquine is a serious and irreversible ophthalmic problem. Clinicians should perform funduscopy to screen for retinopathy before using hydroxychloroquine and identify risk factors behind drug toxicity as early as possible. The appropriate dose (the recommended dose of ≤ 5 mg/kg actual body weight) can minimize the patient's severe retinal damage.^[2,14]^ Hydroxychloroquine remains an effective drug for the treatment of many diseases, but the associated risks need to be recognized.

## Acknowledgments

We would like to thank the members and staff of the Department of Ophthalmology of the Zhuzhou Central Hospital who contributed to this case.

## Author contributions

**Conceptualization:** Gang Wang, Ning Zhuo.

**Formal analysis:** Gang Wang, Ning Zhuo.

**Methodology:** Wei Qi.

**Resources:** Gang Wang, Zheng Liao.

**Supervision:** Feng Tian, Zhenhua Wen.

**Validation:** Feng Tian, Zhenhua Wen.

**Writing – original draft:** Gang Wang.

**Writing – review & editing:** Jingyang Li.
